# Laponite/amoxicillin-functionalized PLA nanofibrous as osteoinductive and antibacterial scaffolds

**DOI:** 10.1038/s41598-022-10595-0

**Published:** 2022-04-21

**Authors:** Zahra Orafa, Hadi Bakhshi, Samira Arab-Ahmadi, Shiva Irani

**Affiliations:** 1 Department of Biology, Science and Research Branch, Islamic Azad University, Tehran, Iran; 2 Department of Life Science and Bioprocesses, Fraunhofer Institute for Applied Polymer Research, Geiselbergstraße 68, 14476 Potsdam, Germany

**Keywords:** Developmental biology, Stem cells, Health care

## Abstract

In this study, Amoxicillin (AMX) was loaded on laponite (LAP) nanoplates and then immobilized on the surface of electrospun polylactic acid (PLA) nanofibers to fabricate scaffolds with osteoinductive and antibacterial activities. The highest loading efficiency (49%) was obtained when the concentrations of AMX and LAP were 3 mg/mL and 1 mg/mL, respectively. FTIR and XRD spectroscopies and zeta potentiometry confirmed the successful encapsulating of AMX within LAP nanoplates. The immobilization of AMX-loaded LAPs on the surface of PLA nanofibers was verified by SEM and FTIR spectroscopy. In vitro release study showed a two-phase AMX release profile for the scaffolds; an initial burst release within the first 48 h and a later sustained release up to 21 days. In vitro antibacterial tests against *Staphylococcus aureus* and *Escherichia coli* presented the ability of scaffolds to inhibit the growth of both bacteria. The biocompatibility assays revealed the attachment and viability of human bone marrow mesenchymal stem cells (hBMSCs) cultured on the surface of scaffolds (*p* ≤ 0.05). The increased ALKALINE PHOSPHATASE (ALP) activity (*p* ≤ 0.001), calcium deposition, and expression of *ALP* and OSTEONECTIN genes indicated the osteoinductivity of functionalized scaffolds for hBMSCs. These LAP/AMX-functionalized scaffolds might be desirable candida for the treatment of bone defects.

## Introduction

Infection control is one of the leading clinical issues related to regeneration surgery in bone tissue engineering^1^. An ideal bone scaffold will presumably be multifunctional, combining different functionalities to simultaneously promote bone regeneration while also limiting microbial infection^2,3^. Replacing traditional manners such as systemic antibiotic administration with the incorporation of antibiotics in scaffolds can be an effective treatment for bone infections^2,3^. Amoxicillin (AMX) is a semisynthetic, broad-spectrum antibiotic that quickly penetrates cell membranes and inhibits bacterial attachment to periodontal ligament cells^4^.

In recent years, silicate-based nano-clays are emerging as bioactive materials and vehicles in drug delivery systems^5–7^. Laponite (LAP) is a synthetic smectite nano-clay with a chemical formula Na_0.7_^+^[(Si_8_Mg_5.5_Li_0.3_)O_20_(OH)_4_]_0.7_^−^ and a disk-shaped structure with a diameter of 25 nm and a thickness of 1 nm^8^. LAP slowly degrades into non-toxic products such as Na^+^, Li^+^, Mg^2+^, and Si(OH)_4_^9,10^, which can promote the proliferation and osteodifferentiation of stem cells without any external osteoinductive factors^5–7,11^. The edges of LAP nanoplates have a positive charge, while their surfaces have a negative charge^8,12^. These charges enable the LAP nanoplates to load with polar molecules and drugs^13,14^.

Polylactic acid (PLA) is a biodegradable and biocompatible polyester, approved by the Food and Drug Administration (FDA), which is commonly employed for the fabrication of nanofibers in bone tissue engineering due to its proper mechanical properties^15^. The main disadvantage of PLA as a biomaterial is the poor surface wettability that causes the lack of cell attachment and proliferation on its surface^16^. Therefore, physical treatments or chemical modifications such as chemical functionalization with bioactive material have neem used to create desirable surface properties for PLA biomaterials^17^.

The functionalization of LAP on the surface of PLA nanofibers can cause osteodifferentiation in human bone marrow mesenchymal stem cells (hBMSCs) without any external osteoinductive factors^7^ In this study, to provide antibacterial activity as well as osteoinductivity in the scaffolds, AMX was encapsulated within LAP nanoplates. The AMX-loaded LAPs (LAP/AMX) were then immobilized on the surface of electrospun PLA nanofibers through crosslinking with polyethylene oxide (PEO). After studying the functionalization process, the features of the fabrication scaffold were investigated regarding their ability to induce proliferation, osteodifferentiation, and antibacterial activity without using any chemical cues.

## Results and discussion

Improving tissue restoration and preventing bacterial infection are favorable properties for bone biomaterials^2,3^. The prior study showed that LAP-functionalized PLA nanofibers could significantly enhance the proliferation and osteodifferentiation of hBMSCs without any external osteoinductive factors^7^. Here, the LAP nanoplates were loaded with different concentrations of AMX before immobilization on the surface of PLA nanofibers to fabricate advanced osteoinductive and antibacterial scaffolds.

### Loading of AMX on LAP nanoplates

LAP nanoplates with a large surface area (330 m^2^/g) high negative charge (− 39.5 mV for LAP suspension, 1 wt%) can encapsulate positively charged drugs and release them as a result of the diffusion or de-intercalation process^18^. In this study, LAP nanoplates were loaded with AMX through mixing in aqueous solutions containing different concentrations of AMX, i.e. 1, 2, and 3 mg/mL, resulting in AMX-loaded LAPs named LAP/AMX1, LAP/AMX2, and LAP/AMX3, respectively. AMX as a semi-synthetic oral antibiotic from the beta-lactam family was selected due to its usage to treat a wide range of gram-positive and gram-negative bacterial infections. The concentration of LAP was kept as low as 3 mg/mL for all experiments since higher concentrations could decrease the loading efficiency of AMX^13,14^. The reason could be the aggregation of LAP nanoplates at high concentrations that reduces the accessibility of their interlayer space for AMX molecules^13,14^.

**Figure 1:**
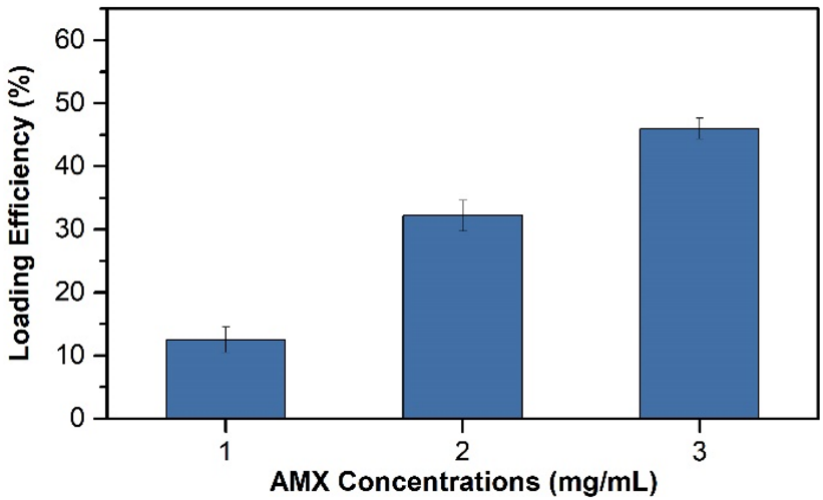
Loading efficiency of AMX in LAP nanoplates at different concentrations.

LAP nanoplates remain connected after dispersion in water and the space between the LAP layers is suitable for the encapsulation of drugs such as AMX^13,14^. The sodium ions in the LAP interlayer space act as ion exchangers for replacing with AMX molecules^13^. To evaluate the loading efficiency of AMX within LAP, the amounts of the unloaded AMX in the solutions were determined through ultraviolet (UV) spectrometry. The loading efficiency was improved from 12 to 49% by increasing the concentration of AMX from 1 to 3 mg/mL (Fig. 1). Since the concentration of LAP was kept constant as 3 mg/mL for all experiments, employing higher concentrations of AMX, which are near to the saturated AMX solubility (2.46 mg/mL^19^ or 3.36 mg/mL^20^), increased the chance for intercalating AMX molecules with the solid LAP nanoplates. The encapsulation of AMX in LAP nanoplates is primarily through the placement of AMX molecules in the space between the LAP layers, however, a small portion of AMX could be adsorbed on the surface of LAP nanoplates due to hydrogen bonding or other weak forces^13^.

Wang et al*.*^13^ reported that the LAP load efficiency for AMX improved by increasing the AMX concentration and reached 10% when the concentration of AMX and LAP were 2 and 3 mg/mL, respectively. They mentioned that this value might not be the highest loading efficiency and further adjustments of the LAP and AMX concentrations and pH are necessary to achieve the maximum loading efficiency^13^. Peng et al*.*^14^ found that at a LAP concentration of 3 mg/mL, the LAP loading efficiency for tetracycline was improved from 8 to 90% by increasing the AMX concentration from 0.1 to 32 mg/mL. However, the encapsulation efficiency was saturated (85.5%) at a tetracycline concentration of 18 mg/mL, meaning no more tetracycline molecules could be loaded on LAP nanoplates at concentrations above 18 mg/mL^14^.

To better investigate the AMX encapsulation between the LAP layers, zeta potentiometry was done. LAP and AMX showed zeta potentials of − 39.9 and + 6.8 mV, respectively. These opposite charges facilitated the intercalation of AMX molecules and LAP nanoplates. After AMX loading, the surface charge of LAP nanoplates decreased, where LAP/AMX1, LAP/AMX1, and LAP/AMX1 presented zeta potentials of − 34.01, − 34.05, and − 34.2 mV, respectively.

**Figure 2:**
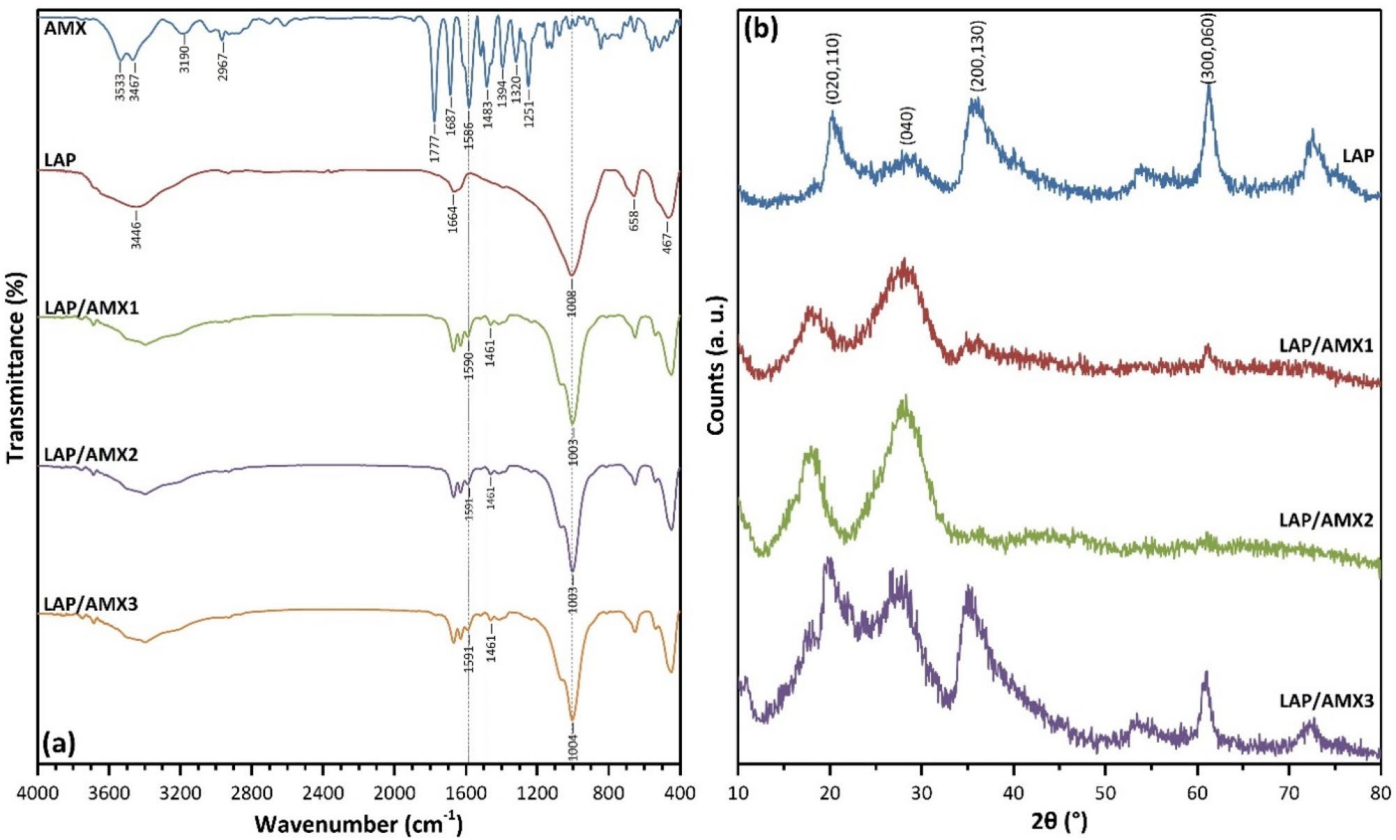
FTIR (**a**) and XRD (**b**) spectra of AMX, LAP, and AMX-loaded LAPs.

The encapsulation of AMX within LAP nanoplates was further evaluated using Fourier transform infrared (FTIR) and X-ray diffraction (XRD) spectroscopy. The FTIR spectrum of AMX showed peaks at 1777 (C=O, β-lactamic, ν), 1687 (C=O, amide, ν), 1586 (C=O, carboxylic, asymmetric ν), 1483 (N‒H, amide), and 1394 cm^−1^ (C=O, carboxylic, symmetric ν) (Fig. 2a)^13,21^. The FTIR spectrum of LAP presented peaks at 3440 (O‒H, ν, absorbed H_2_O), 1664 (O‒H, δ), 1008 (Si‒O, ν), 658 (O–H, δ, Mg_3_OH), and 467 cm^−1^ (Si‒O‒Mg, δ and Si‒O‒Si, δ)^5–7^. The comparison of the FTIR spectra of AMX-loaded LAPs with that of LAP indicated the appearance of two new peaks at 1590 and 1461 cm^−1^ attributing to the encapsulated AMX molecules between LAP layers (Fig. 2a).

The encapsulation of AMX molecules within the LAP interlayer region can result in a shift in layer separation. To investigate the distance between LAP layers before and after AMX encapsulation, XRD spectroscopy was used. (Fig. 2b). The diffraction angle of (020,110), (004), and (200,130) planes shifted from to lower angles for AMX-loaded LAPs, compared to LAP, meaning their interplanar space was increased (Table 1) due to the presence of encapsulated AMX molecules. The changes in diffraction angles and interplanar space values were more significant than the values reported by Wang et al*.*^13^ due to the higher loading efficiency of AMX, *i.e.* 49% compared to 10%, respectively. The FTIR and XRD results agreed with the loading efficiency and zeta potential data, indicating that AMX was successfully loaded into LAP nanoplates.

**Table 1:** Diffraction angles and interplanar space values from XRD spectroscopy.

	Diffraction angle (2θ, °)				Interplanar space (d, Å)			
	(020,110)	(004)	(200,130)	(060, 330)	(020,110)	(004)	(200,130)	(060, 330)
LAP	20.16	27.81	36.11	61.26	4.30	3.20	2.48	1.58
LAP/AMX1	18.11	28.11	34.86	61.22	4.89	3.18	2.57	1.52
LAP/AMX2	18.46	28.26	36.71	61.16	4.81	3.15	2.44	1.53
LAP/AMX3	19.76	26.86	35.01	61.01	4.48	3.31	2.65	1.53

### Immobilizing of AMX-loaded LAP on PLA nanofibers

Through crosslinking with PEO, AMX-loaded LAPs were fixed on the surface of PLA nanofibers. For this purpose, PLA nanofibers were immersed in an aqueous solution containing PEO and LAP or AMX-loaded LAPs separately and dried at room temperature and later at 45 °C under vacuum, which resulted in the physical crosslinking of high-molecular-weight PEO chains (M_v_ = 10^6^ g/mol) with the exfoliated LAP nanoplates^7^. Due to hydrogen bonding and ionic interaction, the surface of LAP nanoplates provides an interface on which the PEO chains can be physically absorbed, resulting in the establishment of links between the PEO chains and, ultimately, the formation of the LAP/PEO network^7^. Since LAP nanoplates are synthetic polyols, they bind to PEO chains as multifunctional crosslinkers^22^. The dipole–dipole interactions between the PLA ester groups and the PEO ether groups could result in adhesion between the LAP/AMX/PEO layer and PLA nanofibers^23^. The functionalized nanofibers were named according to the type of AMX-loaded LAP, i.e., PLA@LAP, PLA@LAP/AMX1, PLA@LAP/AMX2, and PLA@LAP/AMX3.

**Figure 3:**
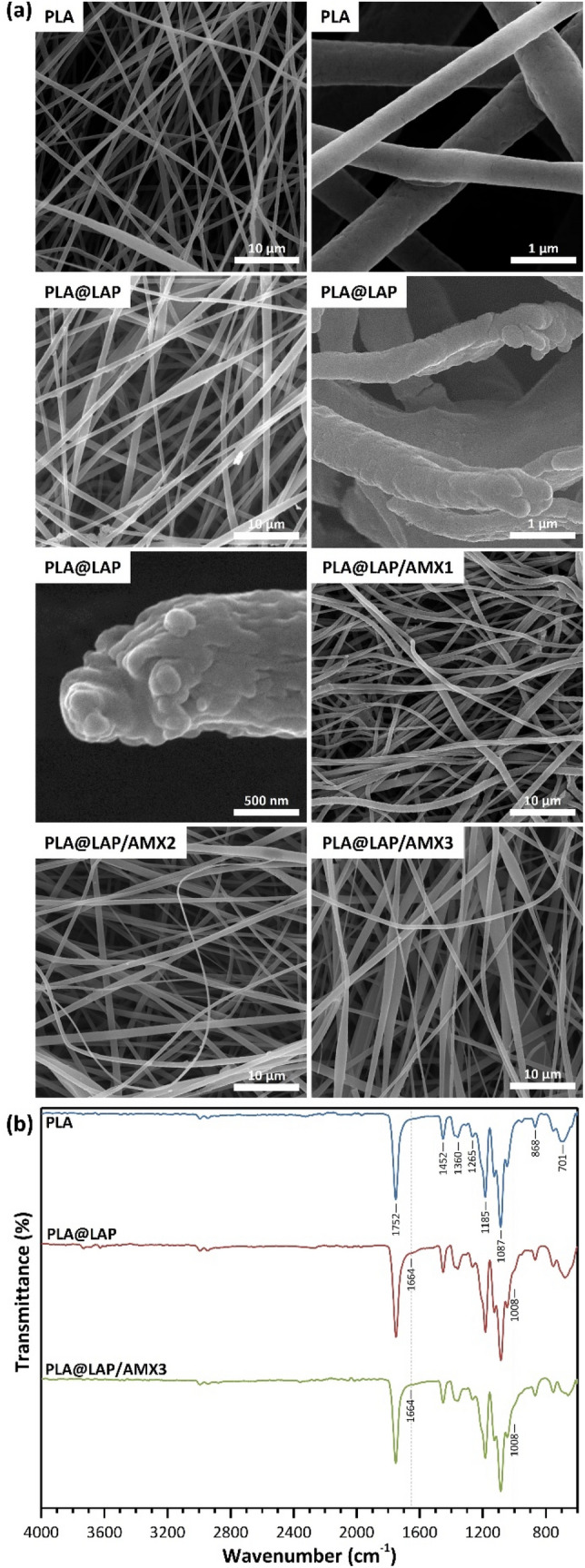
SEM images (**a**) and FTIR spectra (**b**) of scaffolds.

Scanning electron microscopy was used to examine the surface morphology of the nanofibers before and after functionalization (SEM, Fig. 3a). The PLA nanofibers had a smooth surface and a diameter of 610 nm on average. The functionalized PLA nanofibers displayed rough surfaces attributing to the LAP/AMX/PEO layers and higher average dimeters as 980 ± 290, 920 ± 280, 940 ± 310, and 950 ± 320 nm for PLA@LAP, PLA@LAP/AMX1, PLA@LAP/AMX2, and PLA@LAP/AMX3 nanofibers, respectively. The thickness of the LAP/AMX/PEO layers can be calculated as 185, 155, 165, and 170 nm, respectively. Energy-dispersive X-ray (EDX) spectroscopy was used to determine the chemical composition of the nanofibers' surfaces (Table 2). The identical elements of LAP (Si, Mg, and Na) were found on all functionalized nanofibers, while the identical elements for AMX (C, N, and S) were identified only on PLA@LAP/AMX1, PLA@LAP/AMX2, and PLA@LAP/AMX3 nanofibers. The SEM and EDX results confirmed the successful immobilizing of the LAP/AMX/PEO layer on the surface of PLA nanofibers.

**Table 2:** Elemental composition of the surface of scaffolds obtained by EDX spectroscopy.

Scaffold	C (wt%)	O (wt%)	Na (wt%)	Mg (wt%)	Si (wt%)	N (wt%)	S (wt%)
PLA@LAP	50.05	37.82	3.75	4.43	3.95	0.00	0.00
PLA@LAP/AMX1	60.80	19.75	1.21	1.63	2.11	3.84	10.67
PLA@LAP/AMX2	52.68	26.19	1.75	2.37	2.29	3.55	11.17
PLA@LAP/AMX3	50.58	26.97	1.99	2.34	2.17	3.75	12.20

FTIR spectroscopy was used to evaluate the immobilization of AMX-load LAPs on the surface of PLA nanofibers (Fig. 3b). The FTIR spectrum for PLA nanofibers presented peaks at 2994 (C‒H, ν), 1752 (C=O, ν), 1360 and 1452 (C‒H, δ), and 1087 and 1185 cm^−1^ (C‒O, ν)^7^. When the FTIR spectra of functionalized PLA nanofibers were compared to those of PLA nanofibers, two additional peak shoulders appeared at 1664 and 1008 cm^−1^, which were attributed to the immobilized LAP/AMX/PEO layer on the surface of PLA nanofibers, confirming the immobilization process' effectiveness.

### AMX release from scaffolds

**Figure 4:**
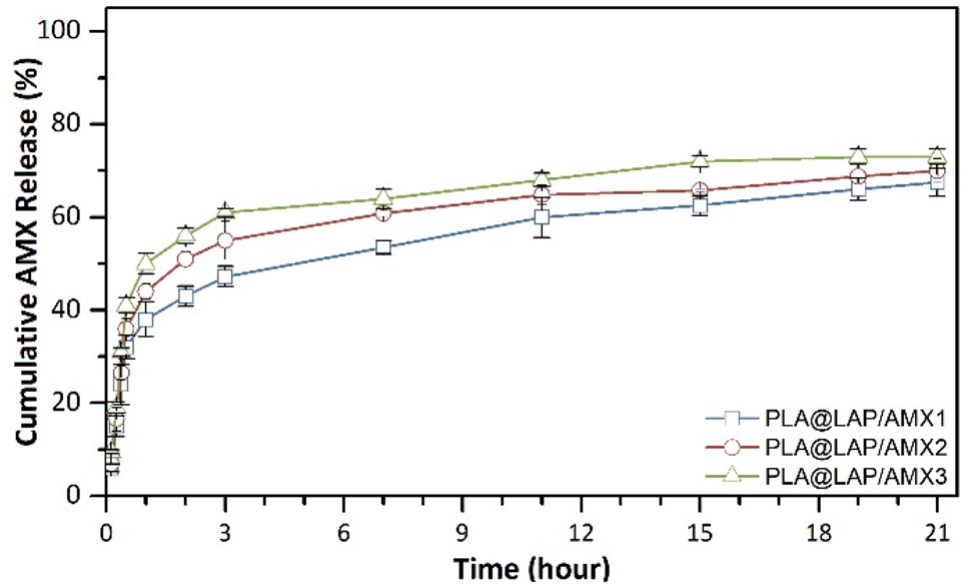
In vitro release of AMX from scaffolds in PBS (1X or 0.01 M, pH 7.4) at 37 °C.

Drugs loaded on LAP nanoplates are released into the environment through a controlled ion-exchange mechanism^24^. The in vitro release of AMX from the scaffolds in phosphate-buffered saline (PBS, 1X or 0.01 M, pH 7.4) at 37 °C was investigated for 21 days (Fig. 4). The saturated solubility of AMX in PBS was determined as 2.8 mg/mL. To follow the sink condition, pieces of (2 × 2 cm^2^) PLA@LAP/AMX1, PLA@LAP/AMX2, and PLA@LAP/AMX3 scaffolds were placed in 1, 1.5, and 1.7 mL of PBS at 37 °C. The PBS media were replaced with new ones, and the released AMX concentration was determined using UV spectroscopy at 230 nm. All AMX-functionalized scaffolds had a two-phase release profile, with an initial burst release in the first 48 h and a later sustained release lasting up to 21 days. The initial burst release was quick, resulting in 43–56 percent of loaded AMX being released. As expected, the initial burst release for the PLA@LAP/AMX1 scaffold was lower (43%) than that of the PLA@LAP/AMX3 scaffold (56%) due to the lower AMX loading for LAP/AMX1 (12%) compared to LAP/AMX3 (46%). Nair et al.^25^ reported the same trend for the release of strontium ranelate (SRA) from polycaprolactone/LAP/SRA composite scaffolds. The next sustained release was slow ensuing in 17–24% release up to 21 days.

### In vitro antibacterial activity of scaffolds

**Figure 5:**
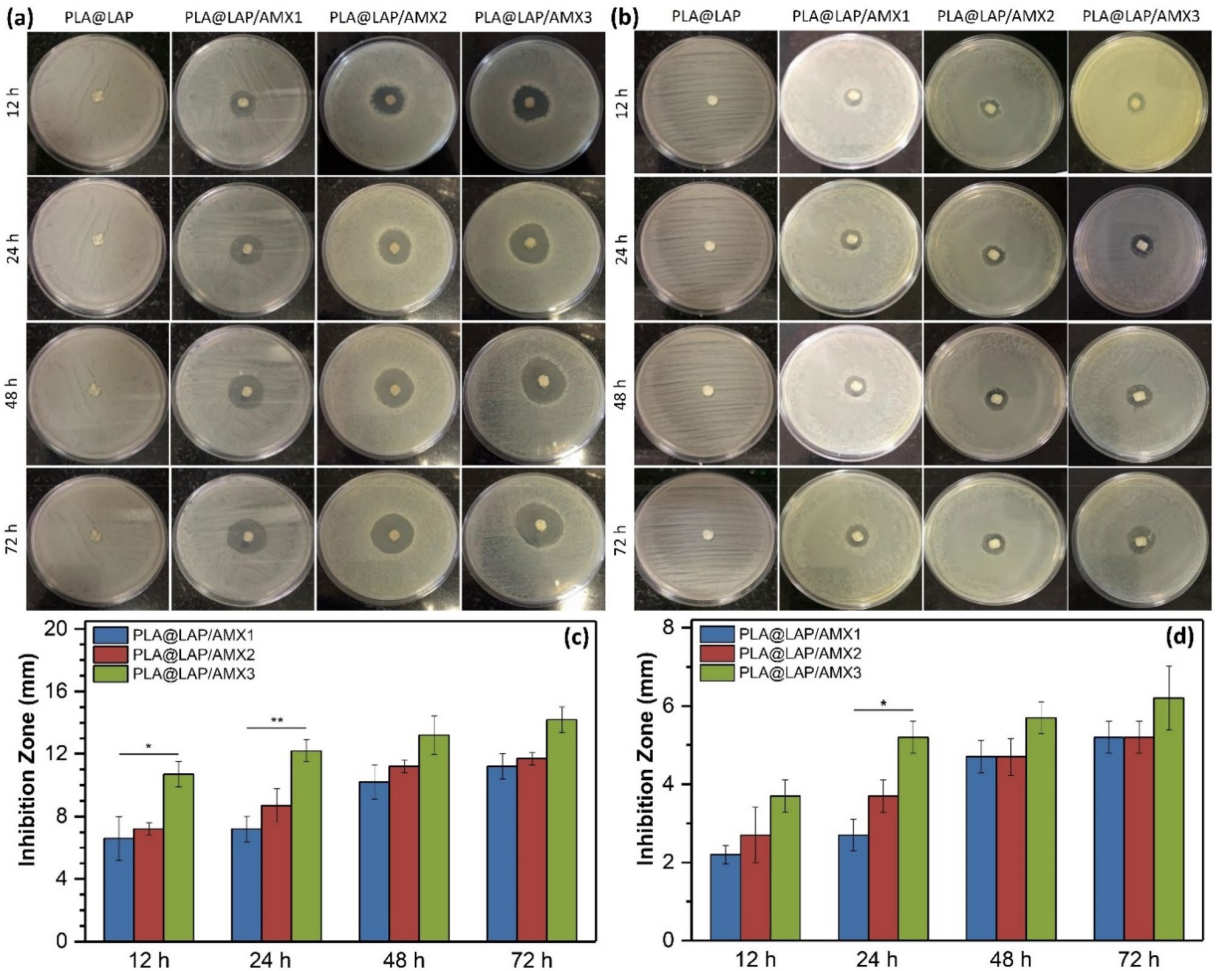
Antibacterial activity of scaffolds against *S. aureus* (**a**) and *E. coli* (**b**). Measured inhibition zone of scaffolds against *S. aureus* (**c**) and *E. coli* (**d**) (n = 3, *p < 0.05, **p < 0.01).

AMX, a beta-lactam antibiotic, inhibits the formation of peptidoglycan, a bacterial cell wall component. AMX interacts with the penicillin-1 binding protein (PBP 1A), an enzyme required for the synthesis of bacterial cell walls, and opens the enzyme's beta-lactam loop and acylated transpeptidase C-terminal domain^26^. This irreversible binding inactivates this enzyme, resulting in a lack of peptidoglycan synthesis, increased cell wall permeability, and ultimately cell lysis and bacterial death^26^. To evaluate the maintenance of AMX activity after the encapsulation and immobilization the in vitro antibacterial activity of the scaffolds was also investigated against *Staphylococcus aureus* (*S. aureus*) and *Escherichia coli* (*E. coli*) as model gram-negative and gram-negative bacteria, respectively, through disk diffusion assay (Fig. 5). After 12–72 h of incubation, the PLA@LAP scaffold did not show an inhibit zone against the development of both bacteria, indicating that it had no antibacterial activity. In contrast, all AMX-functionalized scaffolds showed distinguish inhibit zones again both *S. aureus* and *E. coli* bacteria, where the inhibition zones for the PLA@LAP/AMX3 scaffold releasing higher AMX (Fig. 4) were bigger than those for PLA@LAP/AMX1 and PLA@LAP/AMX2 (Figs. 5c,d). The inhibition zones against *S. aureus* were bigger than those for *E. coli*. The variation in cell membrane architecture between *S. aureus* and *E. coli* bacteria could explain this observation. The multilayered cell envelope structure and more hydrophilic nature of Gram-negative *E. coli* bacteria can better resist antibiotics^27–29^. The presence of an inhibitory zone in a solid medium demonstrated that the loaded AMX could be swiftly released from the scaffolds. Wang et al*.*^13^ reported similar inhibition zones for PLGA nanofibers functionalized with AMX-loaded LAPs against *S. aureus*, where the antibacterial activity of the nanofibers was higher than that of free antibiotics.

### Biocompatibility of scaffolds

**Figure 6:**
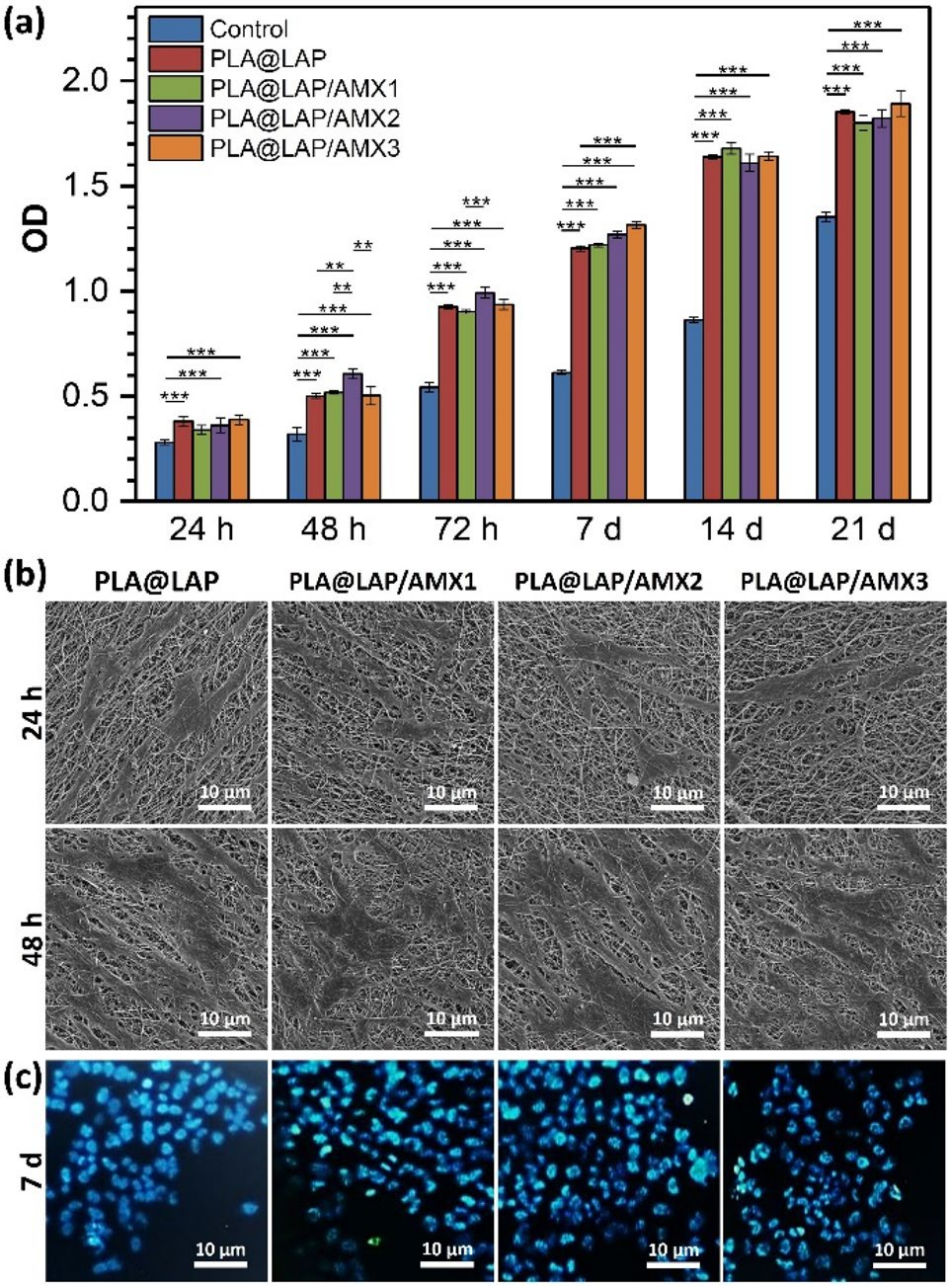
(**a**) Viability of hBMSCs cultured on scaffolds obtained from MTT assay (n = 3, *p < 0.05, **p < 0.01, ***p < 0.001). A tissue culture plate was used as the control. (**b**) SEM images of hBMSCs cultured on scaffolds. (**c**) Fluorescent microscope images of the DAPI-stained hBMSCs cultured on scaffolds.

Scaffolds for tissue engineering must be biocompatible meaning to be able to support the normal cellular activities of the target tissue including signaling and cellular communication and do not stimulate the immune system. The scaffolds' biocompatibility was determined by culturing hBMSCs on them for 21 days and using the MTT test to determine cell viability (Fig. 6a). When compared to a control, i.e. a tissue culture plate without any scaffold, all functionalized scaffolds were able to retain considerably greater cell viability for hBMSCs during the whole 21-day incubation period. (*p* ≤ 0.05). This observation could be due to the high surface hydrophilicity of the functionalized scaffolds arising from LAP and PEO moieties. The cell viabilities for scaffolded functionalized with AMX-loaded LAP were similar to those for unloaded LAP meaning that the released AMX did not generate any cytotoxic effect.

Under SEM, the attachment of hBMSCs to the surface scaffolds was seen (Fig. 6b). After 12 h of incubation, the hBMSCs were adhered to the surface of all functionalized scaffolds and spread across them like an extracellular matrix (ECM), with well-developed cell–cell and cell–matrix connections. The healthiness of the seeded hBMSCs’ nuclei after 7 days of incubation was evalöuated through DAPI staining (Fig. 6c). The hBMSCs cultured on the surface of all functionalized scaffolds had healthy nuclei and DNA content.

These findings showed that LAP/AMX-functionalized scaffolds have high biocompatibility. The Mg^2+^ ions released from LAP nanoplates can interact with the integrin family proteins, *i.e.* fibronectin receptor α5β1 and β1 integrins, and improve the attachment and viability of cells on the LAP/AMX-functionalized scaffolds^30,31^. Wang et al*.*^13^ reported no cytotoxicity for porcine iliac artery endothelial cells (PIECs) seeded on PLGA nanofibers functionalized with AMX-loaded LAPs. Zheng et al*.*^32^ reported no lack of biocompatibility for PLGA nanofibers functionalized with AMX-loaded nano-hydroxyapatite (n-HA).

### Osteoinductivity of scaffolds

The capacity of LAP nanoplates to induce osteodifferentiation is impressive^11^. The Mg^2+^ and Si^4+^ ions being released from LAP nanoplates are involved in the production of collagen type X and type I, respectively^33^. In the Wnt pathway, the Li^+^ ion suppresses the action of glycogen synthase kinase 3 beta (GSK3B). The Wnt signaling affects osteogenesis by regulating the activity of runt-related transcription factor-2 (RUNX2)^34,35^. RUNX2 causes the expression of osteonectin and alkaline phosphatase (*alp)* genes and consequently the osteodifferentiation^36^. The LAP nanoplates can induce osteodifferentiation to stem cells without using any external osteogenic inducer^5–7^.

**Figure 7:**
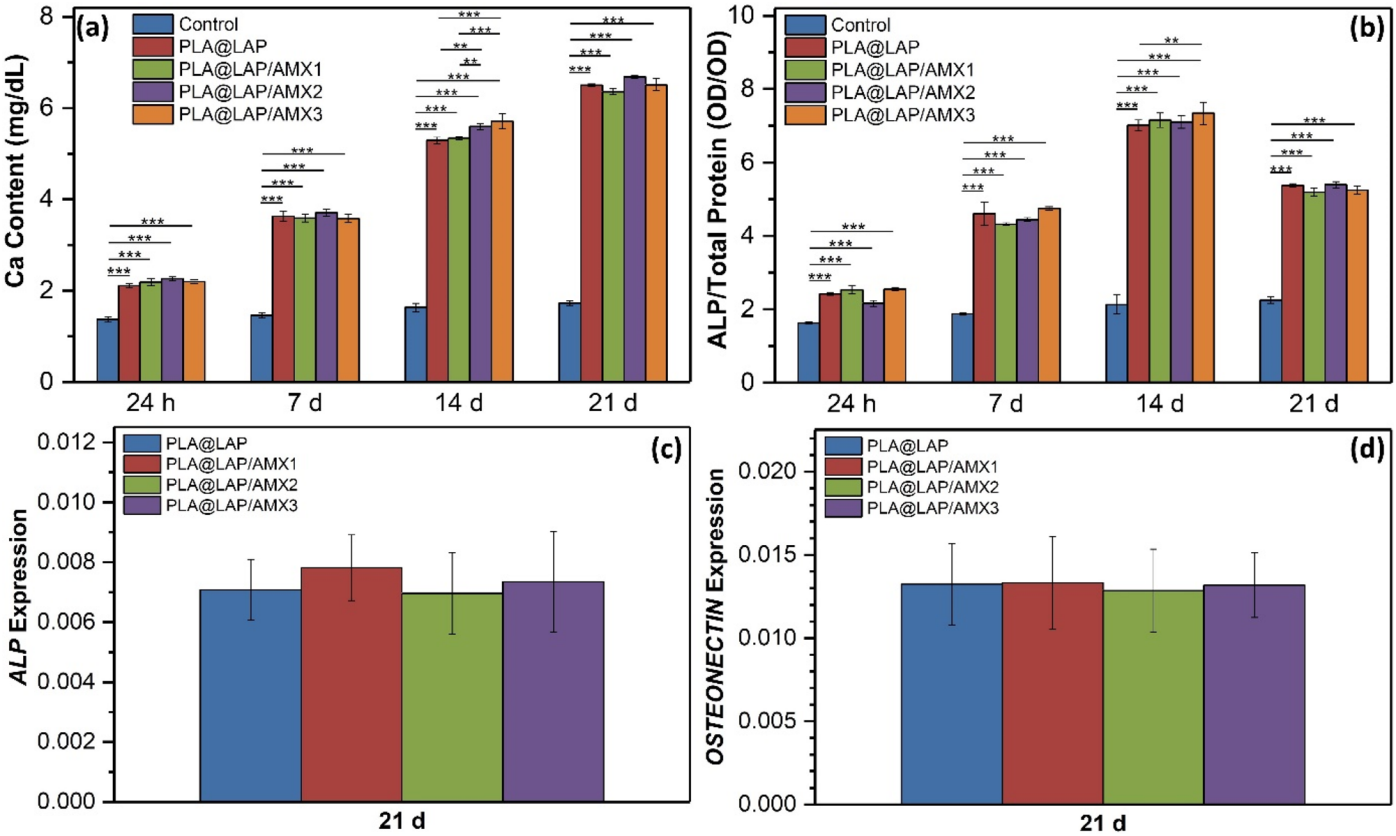
(**a**) Quantity of calcium deposited on the hBMSC-seeded scaffolds determined by calcium CPC kits (n = 3, ***p < 0.01, ***p < 0.001). Tissue culture plate was used as the control. (**b**) ALP activity of hBMSCs cultured on scaffolds (n = 3, ***p < 0.001). Expression of *ALP* (**c**) and *OSTEONECTIN* (**d**) markers in hBMSC cultured on scaffolds (n = 3, *p* > 0.05).

hBMSCs were seeded on the AMX-LAP-functionalized scaffolds and cultured in a non-differential media (without any osteogenic inducer) for 21 days to investigate their osteoinductive impact. The quantity of calcium generated by the osteodifferentiated cells on the scaffolds was measured via calcium content assay (Fig. 7a). During the whole 21-day incubation period, calcium deposition on all functionalized scaffolds grew steadily over time and was significantly higher than the control, i.e. tissue culture plate without any scaffold. (*p* ≤ 0.05). The calcium content values for scaffolded functionalized with AMX-loaded LAP were similar to those for unloaded LAP meaning that the released AMX did not play a negative role in the osteodifferentiation of hBMSCs.

ALP, which is expressed in the early phases of osteodifferentiation, is one of the indicators^37^. This enzyme is involved in the mineralization of the cell matrix and transforms organic pyrophosphate into mineral phosphate^30^. The ALP activity of cells on all functionalized scaffolds was significantly higher than the control during the whole 21 days of incubation (Fig. 7b, p ≤ 0.05). The ALP activity increased gradually over time up to 14 days of incubation and then decreased The ALP activity of rat bone marrow-derived mesenchymal cells (rBMSCs) cultured on gelatin/carboxymethyl chitosan/LAP composite scaffolds^30^ and the ALP activity of the SSEA-4 sub-population of human adipose-derived stem cells (hADSCs) in the presence of various concentrations of LAP nanoplates^38^ were found to be similar.OSTEONECTIN is a calcium-binding glycoprotein that is expressed in mineralized tissues^39–41^. On all functionalized scaffolds, both *ALP* and *OSTEONECTIN* mRNA indicators were expressed in the celled culture for 21 days (Figs. 7c,d). It's worth noting that for cells cultivated on the AMX-loaded scaffolds and the PLA@LAP scaffold, there was no significant change in ALP activity and *ALP* and *OSTEONECTIN* expression levels, which is consistent with calcium content values.

**Figure 8:**
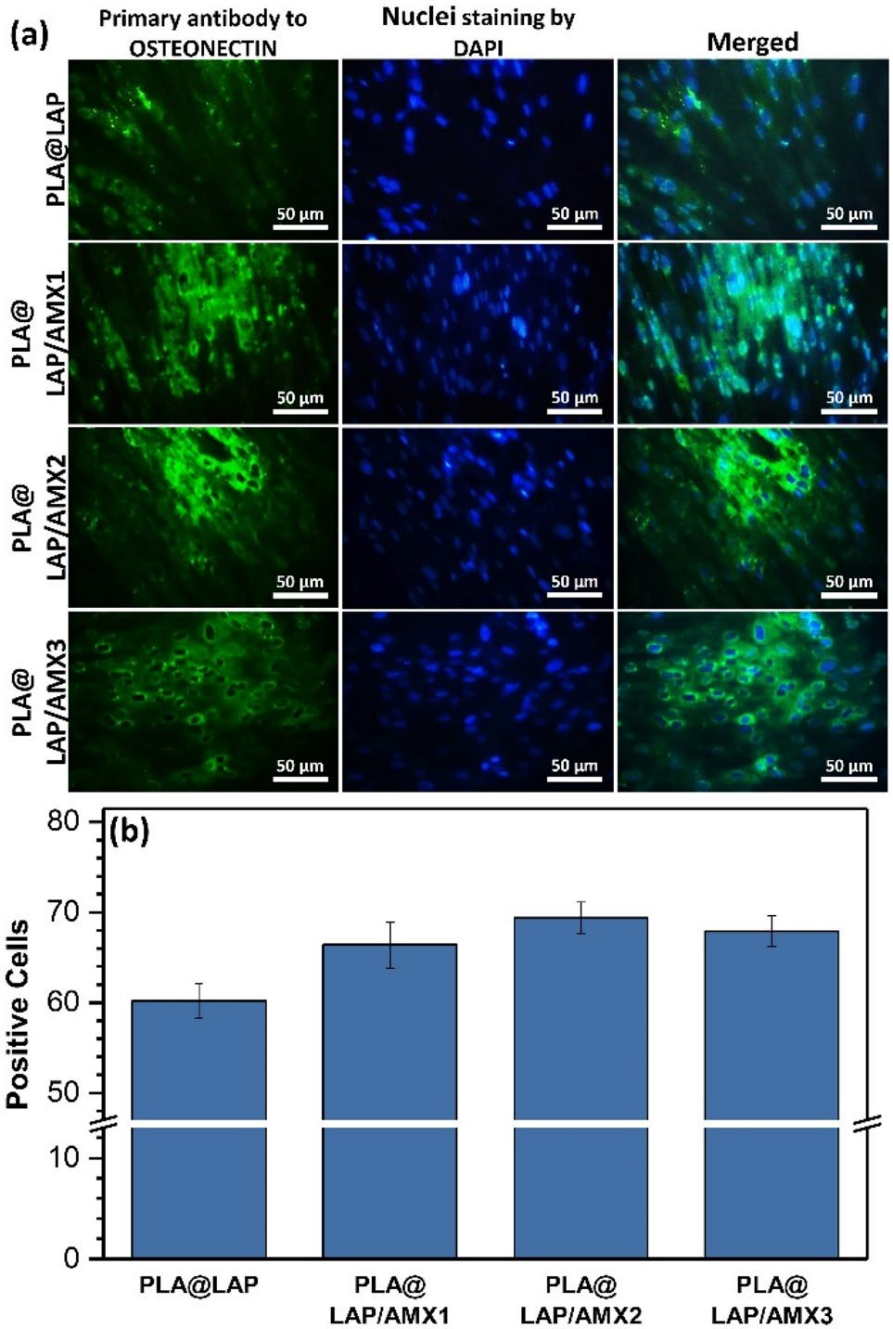
(**a**) ICC images for expression of OSTEONECTIN protein in hBMSCs cultured on scaffolds after 21 days of incubation. (**b**) Quantification of ICC images with Image J software (n = 3).

In the next step, the OSTEONECTIN expression at the protein level was confirmed by ICC assay (Fig. 8).

The semiquantitative data showed no significant differences in OSTEONECTIN expression between cells cultivated on AMX-loaded scaffolds and cells cultured on the PLA@LAP scaffold, indicating that the released AMX did not affect on scaffold osteoinductivity.

## Conclusion

The AMX antibiotic was successfully loaded on LAP nanoplates and then immobilized on the surface of PLA nanofibers. The releasing amount of AMX can be controlled by adjusting the ratio of AMX and LAP in the solution. The functionalized scaffolds displayed high levels of antibacterial activity and biocompatibility, where LAP and PEO moieties provided more hydrophilic surfaces for the attachment and proliferation of hBMSCs. The functionalized scaffolds showed high osteoinductivity for hBMSCs, where the releasing AMX did not play a negative role in the osteodifferentiation. The LAP/AMX-functionalized scaffolds may be applicable as an excellent scaffold for bone tissues engineering to provide a bacteria-free environment for the osteodifferentiation of stem cells without using any external osteogenic inducer.

## Experimental

### Materials

Polylactic acid (PLA, granule, MFR = 6 g/10 min at 210 °C and 2.16 kg), polyethylene oxide (PEO, M_v_ = 10^6^ g/mol), 4ʹ,6-diamidino-2-phenylindole (DAPI) dihydrochloride, 3-[4,5-dimethylthiazol-2-yl]-2,5 diphenyltetrazolium bromide (MTT), glutaraldehyde, hydrochloric acid (HCl) and dimethyl sulfoxide (DMSO) were bought from Sigma-Aldrich (Germany). Chloroform, *N*,*N*-dimethylformamide (DMF), paraformaldehyde, Triton X-100, and Luria Bertani (LB) agar were purchased from Merck (Germany). Laponite RD was supplied by BYK Additives (Germany). Amoxicillin trihydrate (AMX) was provided by the Iran Antibiotic Company (ASICO, Iran). Human bone marrow mesenchymal stem cells (hBMSCs) were obtained from the Pasteur Institute of Iran (Iran). Fetal bovine serum (FBS), bovine serum albumin (BSA), Dulbecco’s modified eagle’s medium (DMEM), trypsin/EDTA solution, and penicillin–streptomycin were received from Gibco (Canada). Phosphate-buffered saline (PBS) solution (1X or 0.01 M, pH 7.4) was purchased from Bio-Idea (Iran).

### Loading of AMX on LAP nanoplates

AMX solutions were obtained by dissolving 10, 20, and 30 mg AMX in 10 mL deionized water separately and stirring for 2 h at room temperature. Then, 30 mg of LAP powder was added to each solution and the LAP/AMX solutions were stirred for 24 h at room temperature. The solutions were centrifuged at 10,621*g* for 10 min. After collecting the supernatants, the precipitated AMX-loaded LAP was redispersed in deionized water and centrifuged at 10,621*g* for 10 min twice to remove the unloaded AMX. All supernatants were collected for measuring the loading efficiency of AMX. The AMX-loaded LAP was freeze-dried (CHRIST, Alpha 1–2 LDplus, Germany) and named LAP/AMX1, LAP/AMX2, and LAP/AMX3 corresponding to the concentrations of AMX solutions of 1, 2, and 3 mg/mL, respectively.

### Electrospinning of PLA nanofibers

Electrospinning of PLA solution(15 wt%) in chloroform/DMF solvent (9/1, v/v) was done by an Asia Nanostructure machine (model CO881007NYI, Iran) at a flow rate of 0.5 mL/h, a voltage of 16 kV, and a needle-to-collector distance of 15 cm on an aluminum foil.

### Immobilizing of AMX-loaded LAP on PLA nanofibers

The PLA scaffolds (0.5 × 0.5 cm^2^) were initially immersed in deionized water/ethanol solution (1/1, v/v) for 3 h at room temperature, washed three times with a large amount of deionized water, and dried for 24 h at room temperature. LAP or AMX-loaded LAPs (6 mg) were separately added to deionized water (0.5 mL) containing PEO (6 mg) and stirred for 24 h at room temperature. The scaffolds were immersed in the obtained solutions for 1 h at room temperature, removed from the solutions, dried at room temperature for 24 h, and again dried in a vacuum oven at 45 °C for 24 h. Finally, the scaffolds were washed three times with deionized water and dried in a vacuum oven at 45 °C for 24 h. The functionalized scaffolds were named according to the immobilized nanoplates on them, i.e. LAP, LAP/AMX1, LAP/AMX2, and LAP/AMX3, as PLA@LAP/AMX1, PLA@LAP/AMX2, and PLA@LAP/AMX3, respectively.

### Instruments

The zeta potential of samples was measured on a Malvern zeta-sizer (ZEN3600, UK) at 633 nm. The samples were dispersed in deionized water using vortexing (20 min) and ultrasonication (10 min) before measurements.

Fourier transform infrared (FTIR) spectroscopy was done on a Nicolet instrument (Avatar, USA) with a wavenumber range of 400–4000 cm^−1^ to follow the chemical groups of samples.

The crystalline structure of samples was analyzed by X-ray diffraction (XRD) spectroscopy (Philips, Netherlands) in the range (2θ) of 10°–70° using Cu Kα radiation (λ = 1.54 Å) at 40 kV and 200 mA. The interplanar space (d) was calculated by Bragg's law:^13^

$$\mathrm{d}= \frac{\uplambda }{2\mathrm{ sin\theta }}$$

where λ is the wavelength of the copper anode source (1.54 Å) and θ is the diffraction angle of each diffraction plane.

Ultraviolet (UV) spectrometry was done on a PG instrument (T80, Australia) at a wavelength of 230 nm using a standard curve of the absorption of AMX solutions (0.01–0.08 mg/mL) in deionized water.

The surface morphology and elemental composition of nanofibers were studied through field emission scanning electron microscopy (FE-SEM, Mira3, Tescan, Czech Republic) and energy-dispersive X-ray (EDX) spectroscopy, respectively. The average diameter of nanofibers was measured employing Image J software (version 1.53a).

### Methods

For determining the loading efficiency of AMX in LAP nanoplates, the concentration of unloaded AMX in supernatants was measured by UV spectrometry at 230 nm. The loading efficiency was calculated according to the following equation:

$$\text{Loading efficiency }= \frac{{\mathrm{M}}_{\mathrm{t}}}{{\mathrm{M}}_{0}}\times 100\mathrm{\%}$$

where M_t_ is the mass of encapsulated AMX and M_0_ is the initial amount of AMX^13^.

To study the in vitro AMX release, scaffolds (2 × 2 cm^2^) were incubated in 1–1.7 mL of PBS (1X or 0.01 M, pH 7.4) at 37 °C for 21 days. Every 3 days, the PBS media were replaced with fresh ones and the concentration of the released AMX was measured by UV spectrometry at 230 nm. The cumulative AMX release was calculated according to the following equation:

$${\text{Cumulative drug release }\left(\mathrm{\%}\right)}_{\mathrm{t}}={\left(\frac{{M}_{t}}{{M}_{0}}\times 100\right)}_{t}+{\left(\mathrm{drug \, release},\mathrm{ \% }\right)}_{t-1}$$

M_t_ is the cumulative amount of the released AMX at time t, and M_o_ is the initial amount of the loaded AMX.

### Disk diffusion assay

The In vitro antibacterial activity of the scaffold was evaluated through the disk diffusion method using *Staphylococcus aureus* (*S. aureus*) and *Escherichia coli* (*E. coli*) as model bacteria. LB agar plates were prepared, by dissolving LB agar powder (38 g) in distilled water (1000 mL), sterilizing in an autoclave at 121 °C and 1.2 atm for 15 min, pouring into sterile plates, and cooling at room temperature. One colony of each bacterium was dispersed in physiological saline (5 mL) and spread on LB agar plates separately using sterile cotton swabs. The scaffolds (0.5 × 0.5 cm^2^) were placed on the center of each plate and incubated at 37 °C for 72 h. Finally, the inhibition zone was calculated according to the following equation:

$${\text{Inhibition zone }} \left( {{\text{mm}}} \right) = \left( {{\text{diameter of inhibition circle}} {-} {\text{diameter of scaffold}}} \right)/2$$

### Cell seeding on scaffolds

hMSCs were cultured in a DMEM medium containing FBS (10%) and penicillin–streptomycin (1%) in a humidified CO_2_ (5%) incubator at 37 °C. After the third passage, the cells were trypsinized with a Trypsin/EDTA solution, suspended in the culture medium, seeded on the scaffolds in 96-well tissue culture plates (1 × 10^4^ cells/well) in triplicate, and incubated at 37 °C for 30 min. Later, 170 μL of the culture medium was added to each well and the plates were incubated at 37 °C for 21 days. hMSCs were cultured onto the tissue culture plate without any scaffold as control.

### Biocompatibility assays

The viability of hBMSCs seeded on scaffolds for 1, 2, 3, 7, 14, and 21 days of incubation was determined through MTT assay. The seeded scaffolds were washed with PBS (1X or 0.01 M, pH 7.4) and incubated with MTT solution (5 mg/mL, 200 μL) for 3 h. After replacing the MTT solution with DMSO (120 μL), the plates were incubated at 37 °C for 30 min and shaken for 15 min. Eventually, the optical density (OD) of the DMSO solutions was measured at 570 nm using a microplate reader (Awareness Technology Inc., USA).

Under FE-SEM, the seeded cells' adhesion to the scaffolds after 12 and 48 h of incubation was evaluated. The seeded cells were fixed with a glutaraldehyde solution (2.5%), then dehydrated using an ethanol gradient series (30, 50, 70, 80, 90, 95, and 100%), and then dried in a vacuum oven. To examine the healthiness of the seeded cells’ nuclei after 7 days of incubation, DAPI staining was performed. The seeded cells were fixed by paraformaldehyde solution (4%) for 10 min, permeabilized with Triton X100 solution (0.1%) for 2 min, and stained with DAPI for 5 min. The images were taken under a fluorescent microscope (Laborlux D, Leitz, Germany).

### Osteodifferentiation assays

The quantity of calcium deposited by cells on scaffolds after 1, 7, 14, and 21 days of incubation was determined using a calcium content assay kit (Pars Azmoon, Iran). The seeded scaffolds were shaken with HCL solution (0.1 mL, 0.6 N) in microtubes at 4 °C for 45 min. The calcium concentration was measured using a microplate reader at 570 nm according to the manufacturer’s protocol.

The osteodifferentiation of the seeded cells after 1, 7, 14, and 21 days of incubation was detected by assessing the ALKALINE PHOSPHATASE (ALP) activity. The seeded cells were lysed at 4 °C for 45 min using an enzyme extraction buffer (Kia Zist, Iran) and a PROTEASE inhibitor (Kia Zist, Iran), as directed by the manufacturer. The cells were centrifuged for 15 min at 4 °C at 2500 g. An ALP assay kit (Pars Azmoon, Iran) was used to evaluate the supernatant at 405 nm using a microplate reader according to the manufacturer's procedure. The ALP activity values were normalized by the total protein content of the lysate obtained via a total protein assay Kit (Pars Azmun, Iran) using a microplate reader at 546 nm according to the manufacturer’s protocol.

After 21 days of incubation, the expression of the *ALP* and *OSTEONECTIN* genes at the transcription level within the seeded cells was assessed using a real-time polymerase chain reaction (PCR) assay. A total RNA extraction kit was used to extract the total ribonucleic acid (RNA) from the seeded cells. (Pars Tous Biotechnology, Iran) and converted to complementary deoxyribonucleic acid (cDNA) by a cDNA synthesis kit (Pars Tous Biotechnology, Iran) following the manufacturer’s instruction. The quantitative PCR was performed on a thermal cycler (Rotor-Gene 6000, Qiagen, USA) using a master mix reagent (2X Real-Time PCR Master Mix, Biofact Co., South Korea) and SYBER green (Amplicon, Denmark). The primers were purchased from Takapouzist (Iran); *BETA-2 MACROGLOBULIN (B*_*2*_*M)*; F:5ʹ- GCGTACTCCAAAGATTCAG-3ʹ, R:5ʹ-GAGATAGAAAGACCAGTCC-3ʹ; *ALP*; F:5ʹ-GAGTATGAGAGTGACGAG-3ʹ, R:5ʹ-GCCAGACCAAAGATAGAG-3ʹ; *OSTEONECTIN*; F:5ʹ-AGTCATCAAGCCCACCAG-3ʹ, R:5ʹ- TAATAGCCCTCCTCATTA-3ʹ.

The detection of OSTEONECTIN protein in seeded cells after 21 days of incubation was done through immunocytochemistry (ICC). The seeded scaffolds were fixed with paraformaldehyde (4%) at 4 °C for 20 min, permeabilized with Triton X-100 solution (4%), immersed in BSA for 45 min to block the nonspecific binding sites, and incubated with anti-osteonectin primary antibody (Sigma-Aldrich, Germany) at 4 °C overnight, incubated with secondary antibodies (Chemicon Temecula) at 37 °C for 1 h, and stained with DAPI to observe under a confocal fluorescent microscope (Labomed, PCM400, Denmark).

### Statistical analysis

To evaluate the significance level between the studied groups, ANOVA one-way test was done by SPSS software (version 22, SPSS Inc., USA). The *p* values less than 0.05 were considered significant. The results were displayed as * for *p* < 0.05, ** for *p* < 0.01, and *** for *p* < 0.001.

### Ethical approval

hBM-MSCs obtained from the Stem Cell Technology Research Center (Tehran, Iran) were isolated from iliac crest bone marrow aspirates of healthy adult donors after obtaining approval from the Ethics Committee of Tehran University of Medical Sciences (Tehran, Iran).

## Data Availability

All data generated or analyzed during this study are included in the manuscript. The raw data are available from the corresponding author upon reasonable request.
